# Assessing unsafe behaviors and their relationship with work-related factors among EMS staff in Iran: a cross-sectional study

**DOI:** 10.1186/s12873-024-00980-5

**Published:** 2024-04-23

**Authors:** Reza Asadi-JabehDar, Rajab Dashti-Kalantar, Saeid Mehri, Alireza Mirzaei, Aghil Habibi Soola

**Affiliations:** 1 Department of Emergency Nursing, School of Nursing and Midwifery, Ardabil University of Medical Sciences, Ardabil, Iran; 2 Department of Critical Care Nursing, School of Nursing and Midwifery, Ardabil University of Medical Sciences, Ardabil, Iran; 3 Department of Nursing, School of Nursing and Midwifery, Ardabil University of Medical Sciences, Ardabil, Iran

**Keywords:** Unsafe behavior, Stress, Fatigue, Teamwork, Emergency medical services

## Abstract

**Background:**

Emergency Medical Services (EMS) staff often encounter various safety incidents. Work-related factors can lead to unsafe behaviors and safety incidents. This study assessed unsafe behaviors and their relationship with work-related factors among EMS staff.

**Methods:**

This descriptive-correlational study used census sampling method to select 284 EMS staff in Ardabil Province, northwest of Iran, from April to June 2023. The data collection tools were demographic and occupational information form, Mearns Unsafe Behavior Scale, Cohen Perceived Stress Scale, Michielsen Fatigue Scale, and Patterson Teamwork Scale. The data were analyzed using the SPSSv-16, descriptive statistics, Pearson correlation, and multiple linear regression.

**Results:**

The mean of unsafe behavior, fatigue, perceived stress, non-conflict of teamwork, and conflict of teamwork were 15.80 (± 4.77), 20.57 (± 6.20), 16.10 (± 6.13), 117.89 (± 17.24), and 40.60 (± 9.59), respectively. Multiple linear regression analysis showed that “partner trust and shared mental models (PTSMM),” “physical fatigue,” “age,” “type of shift,” “employment status,” and “overtime hours per month” were predictors of general unsafe behavior (*P* < 0.001) and “mild task conflict (MTC),” “employment status,” “partner trust and shared mental models (PTSMM)” were predictors of unsafe behavior under incentives EMS staff (*P* < 0.001).

**Conclusion:**

The present study showed that some work-related factors were predictors of unsafe behaviors. The negative consequences of unsafe behaviors should be considered, and long-term planning should be done to reduce them. Developing specific guidelines for addressing unsafe behaviors, implementing measures to reduce fatigue, managing overtime hours in the workplace, and Establishing a system where novice staff work with experienced staff during their first year can be beneficial in reducing these behaviors among EMS staff.

## Introduction

One of the main parts of healthcare provider organizations is emergency medical services (EMS), which significantly contributes to reducing out-of-hospital deaths and is one of the leading and well-known fields in medical assistance programs today [1, 2]. The conditions of the EMS work environment are very complex and challenging [3]. EMS staff provide services to patients with critical conditions and in complex situations and often encounter various safety incidents while performing their duties [4, 5].

Most safety incidents are attributed to unsafe behaviors, such as failure to follow instructions [6]. Unsafe behaviors can result in fatal injuries primarily caused by ambulance vehicle accidents and non-fatal injuries caused by physical exertion (lifting, carrying, or transferring patients and equipment), exposure to hazardous substances (body fluids and chemicals), and falls [7–9]. A study conducted on emergency medical personnel in Iran found that personal health behaviors were reported to be very good (86.5%) [10]. One study found significant positive interrelationships between work-related factors, unsafe behavior, and safety incident involvement among EMS crew members [11]. Another study reported that a high percentage of EMS personnel displayed potentially safety-compromising behaviors while providing patient care [12]. A survey of healthcare staff emphasized the importance of adhering to safe behaviors and following instructions for use [13].

Studies have shown that various factors contribute to unsafe behaviors, and it is necessary to investigate and recognize the influencing factors to prevent and reduce these behaviors effectively [14, 15]. In the case of EMS staff, their unique workplace, which often involves tending to injured and critically ill patients in need of high-quality healthcare, exposes them to elevated stress levels [16] and fatigue [17].

Stressors like workload pressure can related to unsafe behaviors, reduce safety rule compliance, and cause occupational injuries [18]. In recent decades, stress has emerged as a significant public health concern, with its adverse effects among healthcare workers [19–21]. Stress is often associated with losing control over one’s circumstances and leads to changes in physical, psychological, and emotional structures [20, 22]. An increase in safety incidents and unsafe behaviors has been attributed to the fatigue of EMS staff [12, 23]. Fatigue can be effective in causing or aggravating diseases [24] and significantly hampers an individual’s ability to function effectively and safely [25]. Studies have indicated that reducing fatigue can considerably improve patient and staff safety [23, 26, 27]. Stress and fatigue can influence behavioral performance and teamwork for various reasons [22, 28, 29].

Effective communication between team members and teamwork is effective in achieving a common objective and is significantly associated with lower occurrences of adverse events [30]. In Iran, medical emergency teams have two technicians in each ambulance and usually work 24-hour shifts. With strong and sufficient teamwork, these technicians can make timely decisions in high-casualty missions and busy environments, perform effectively, and provide quality care [31]. Studies have also emphasized the importance of teamwork in enhancing safety and the quality of healthcare services [18, 32].

Unsafe behaviors adversely influence staff and patient safety. It also imposes a substantial economic burden on the healthcare system and society [33, 34]. According to the studies, assessing the relationship between unsafe behaviors of EMS staff and work-related factors such as stress, fatigue, and teamwork is very important [11, 17, 18, 35]. Despite some previous investigations, research on unsafe behaviors and their influencing work-related factors is limited in Iran. As a result, this study assessed unsafe behaviors and their relationship with work-related factors among EMS staff.

## Methods

### Study design

The population for this descriptive-correlational study included the EMS staff in Ardabil province, northwest of Iran, from April to June 2023. The census sampling method was used, and the inclusion criteria included having at least six months of work experience in EMS. Administrative staff and those with incomplete questionnaires were excluded from the study. The EMS in Ardabil has 25 urban bases and 35 roadside bases. Researchers obtained a permit from the University of Medical Sciences Ethics Committee and a recommendation letter from the Vice Chancellor for Research before starting the study. They presented this letter to officials at EMS centers. Then, they visited all EMS centers in Ardabil province. Before sampling, they explained the study’s design and purpose to the participants. The participants were assured that their identities would remain anonymous and all information provided would be kept confidential. Written informed consent was obtained from all participants before distributing the paper version of the questionnaire. Of the 338 EMS staff who met the inclusion criteria, 24 did not consent to participate in the study, 21 questionnaires were not returned, and nine questionnaires were incomplete. Finally, 284 EMS staff were included in the study by completing a questionnaire.

### Data collection tools

Five self-report scales, including the demographic and occupational information form, Mearns Unsafe Behavior Scale, Cohen Perceived Stress Scale, Michielsen Fatigue Scale, and Patterson Teamwork Scale, were adopted for assessment purposes in this study.

### Demographic and occupational information form

Demographic and occupational information form included age, marital status, employment status, work experience, workplace, educational levels, number of missions in 24 h, type of shift, and overtime hours per month.

### Unsafe behavior questionnaire

Unsafe behavior was based on the unsafe behavior scale [36]. Sedlar first used this questionnaire for EMS staff, measuring the extent of safety-compromising behavior due to breaking the rules and using shortcuts. Cronbach’s alpha coefficient was 0.91 [11]. It consists of an 11-item scale where the negatively worded statements related to general unsafe behavior (eight items, e.g., ‘I ignore safety regulations to get the job done’) and unsafe behavior under incentives (three items, e.g., ‘I am under pressure from my workmates to break rules’) are answered on a 5-point scale (from 1 = never to 5 = always). Higher scores represent more frequent unsafe behaviors. After obtaining permission from the original developer, two translators independently translated an English version of this questionnaire into Persian. To determine the content validity index (CVI) and content validity ratio (CVR), the questionnaire was provided to 12 faculty members at Ardabil University of Medical Sciences. The content validity index was assessed separately by experts for each question using three criteria: simplicity, appropriateness, and certainty, based on a four-part spectrum (for example, in terms of simplicity, quite simple, somewhat complex, and complex). After considering all factors, the content validity index was 0.88, indicating a high validity level. Furthermore, the Cronbach’s alpha coefficient was calculated and found to be 0.83, confirming the reliability of the questionnaire.

### Perceived stress questionnaire

The Perceived Stress Scale (PSS) was developed by Cohen et al. within three 4, 10, and 14-item versions to measure perceived stress [37]. The PSS-10 possesses adequate internal consistency, with Cronbach’s alpha coefficients ranging from 0.67 to 0.91 [38–40]. This questionnaire is scored based on a 5-point Likert scale (0 = never, 4 = always). In this research, a 10-item version was used, and items 4, 5, 7, and 8 were scored reversely. The overall score ranged from 0 to 40, with higher scores indicating higher levels of perceived stress. The present study obtained a Cronbach’s alpha of 0.82, indicating good internal consistency.

### Fatigue questionnaire

The Fatigue Assessment Scale (FAS) was developed by Michielsen et al. with a Cronbach’s alpha coefficient of 0.83 [41]. This tool consists of 10 items assessing perceived physical and mental fatigue, with a 5-point Likert-type response scale ranging from 1 = “never” to 5 = “always”. Higher scores show higher levels of fatigue. Except for questions 4 (I have enough energy for everyday life) and 10 (I can focus very well when I am busy doing), which represent positive issues, the remaining eight questions out of 10 were related to negative points. In this study, Cronbach’s alpha was 0.80.

### Teamwork questionnaire

This questionnaire was designed by Patterson et al. with a Cronbach’s alpha of over 0.70 [42]. It comprises 45 items, including nine sub-scales in two conflict (confronting teamwork) and non-conflict (interest in teamwork) groups. The non-conflict sub-scale had 33 items in five sub-sets: team orientation (TO), team structure and leadership (TSL), partner communication, team support and monitoring (PCTSM), partner trust and shared mental models (PTSMM), and partner adaptability and backup behavior (PABUB). The Conflict Scale had 12 items in four sub-sets, including Process Conflict (PC), Strong Task Conflict (STC), Mild Task Conflict (MTC), and Interpersonal Conflict (IC). The responses are rated on a 5-point scale (Disagree, Slightly Disagree, Neither Agree nor Disagree, Slightly Agree, Agree). The non-conflict sub-scale is scored from 0 (disagree) to 4 (agree), and the conflict sub-scale is scored in reverse (0 = agree, 4 = disagree) to reflect the positive nature of reducing conflict. In the current study, Cronbach’s alpha was calculated at 0.86.

### Data analysis

Data analysis was performed using Social Science Statistical Package (SPSS) version 16. Descriptive analysis was used to describe the characteristics of the samples. Pearson correlation was used to investigate the relationship between unsafe behaviors, perceived stress, fatigue, and teamwork of EMS staff. The predictive factors of general unsafe behavior and unsafe behavior under incentives from EMS staff were identified via multiple linear regression analysis.

## Results

The overall response rate of the study was 84% (284). Among the study participants, 56.3% were under 30, and 57.0% were married. Most of the participants (51.1%) were employed. It was found that 63.0% had less than five years of work experience. 66.5% of the participants worked in urban areas, and 63% had at least some college education. Additionally, 43.7% of participants had less than five missions in 24 h. 48.6% had a 12-hour shift in one day, and 53.5% had overtime work between 80 and 120 h during the last working month (Table 1).

**Table 1:** General characteristics of Emergency Medical Services staff (*n* = 284)

Variables	Categories	N	%
Age	21–30	160	56.3
	31–40	83	29.2
	More than 40	41	14.5
Marital status	Single	122	43.0
	married	162	57.0
Employment status	Employed	145	51.1
	Contractual	69	24.3
	Commitment	70	24.6
Work Experience	less than 5	179	63.0
	5–10	65	22.9
	More than 10	40	14.1
Workplace	Urban centers	189	66.5
	Road centers	95	33.5
Educational levels	Diploma	105	37.0
	At least some college	179	63.0
Number of Missions in 24 h	less than 5	124	43.7
	5–8	93	32.7
	More than 8	67	23.6
Type of Shift	12 h	138	48.6
	24 h	116	40.8
	More than 24 h	30	10.6
Overtime hours per month	less than 80	66	23.2
	80–120	152	53.5
	More than 120	66	23.2

Table 2 shows the descriptive statistics and the relationship among unsafe behavior, subgroups of fatigue, perceived stress, and subgroups of teamwork among EMS staff. The mean and standard deviation (SD) for unsafe behavior, fatigue, and perceived stress were 15.80 (4.77), 20.57 (6.20), and 16.10 (6.13), respectively. Also, the mean and standard deviation in teamwork for two groups of conflict (confronting teamwork) and non-conflict (interest in teamwork) were 40.60 (9.59) and 117.89 (17.24).

**Table 2:** Descriptive statistics and correlations among the study variables (*N* = 284)

Variables			Unsafe behavior r (p)
	Mean ± SD	Min / Max	
**Unsafe behavior**	15.80 ± 4.77	11–45	
General unsafe behavior	11.77 ± 3.57	8–30	
Unsafe behavior under incentives	4.02 ± 1.60	3–15	
**Fatigue**	20.57 ± 6.20	10–45	
Physical Fatigue	12.80 ± 4.14	6–29	0.295 (< **0.001**)
Mental Fatigue	7.76 ± 2.68	4–16	0.250 (< **0.001**)
**Perceived Stress**	16.10 ± 6.13	1–35	0.189 (< **0.001**)
**Non-conflict items of teamwork**	117.89 ± 17.24	37–157	
The Team Orientation (TO)	18.77 ± 6.27	6–30	-0.006 (0.918)
The Team Structure and Leadership (TSL)	23.93 ± 5.26	6–30	-0.196 (< **0.001**)
The Partner Communication, Team Support, and Monitoring (PCTSM)	40.53 ± 6.74	10–50	-0.185 (**0.002**)
The Partner Trust and Shared Mental Models (PTSMM)	14.12 ± 4 0.54	7–28	0.312 (< **0.001**)
The Partner Adaptability and Backup Behavior (PABUB)	20.52 ± 4.37	6–25	-0.245 (< **0.001**)
**Conflict items of teamwork**	40.60 ± 9.59	12–61	
The Mild Task Conflict (MTC)	8.21 ± 2.99	3–15	0.071 (0.235)
The Strong Task Conflict (STC)	9.11 ± 3.04	3–15	-0.072 (0.229)
The Process Conflict (PC)	11.52 ± 3.19	3–21	-0.225 (< **0.001**)
The Interpersonal Conflict (IC)	11.75 ± 3.16	3–15	-0.226 (< **0.001**)

Unsafe behavior had a positive correlation with physical fatigue (*r* = 0.295, *p* < 0.001), mental fatigue (*r* = 0.250, *p* < 0.001), perceived stress (*r* = 0.189, *p* < 0.001), and partner trust and shared mental models (PTSMM) (*r* = 0.312, *p* < 0.001). Furthermore, the unsafe behavior had a negative correlation with team structure and leadership (TSL) (*r* = -0.196, *p* = 0.001), partner communication, team support, and monitoring (PCTSM) (*r* = -0.185, *p* = 0.002), partner adaptability and backup behavior (PABUB) (*r* = -0.245, *p* < 0.001), process conflict (PC) (*r* = -0.225, *p* < 0.001), and interpersonal conflict (IC) (*r* = -0.226, *p* < 0.001).

The results of the linear regression analysis for general unsafe behavior and unsafe behavior under incentives of EMS staff based on independent and demographic variables are shown in Table 3.

**Table 3:** Linear regression analysis of general unsafe behavior and unsafe behavior under incentives

Variables	General unsafe behavior			Unsafe behavior under incentives		
	Beta	Sig		Beta	Sig	
(Constant)	*p* < 0.001			*p* < 0.001		
Physical Fatigue	0.176	**0.022**	R^2^ = 0.218 F = 3.477 *p* < 0.001	0.065	0.379	R^2^ = 0.263 F = 4.449 *p* < 0.001
Mental Fatigue	0.046	0.591		0.149	0.074	
Perceived Stress	-0.070	0.346		0.070	0.330	
The Team Orientation (TO)	-0.029	0.648		-0.098	0.106	
The Team Structure and Leadership (TSL)	0.064	0.455		0.037	0.657	
The Partner Communication, Team Support, and Monitoring (PCTSM)	-0.032	0.746		0.157	0.106	
The Partner Trust and Shared Mental Models (PTSMM)	0.228	**0.001**		0.160	**0.019**	
The Partner Adaptability and Backup Behavior (PABUB)	-0.130	0.156		-0.108	0.223	
The Mild Task Conflict (MTC)	0.083	0.273		0.240	**0.001**	
The Strong Task Conflict (STC)	-0.028	0.723		-0.015	0.850	
The Process Conflict (PC)	-0.058	0.547		-0.102	0.277	
The Interpersonal Conflict (IC)	0.046	0.621		-0.089	0.327	
Age	-0.157	**0.024**		-0.125	0.064	
Marital statues	-0.047	0.483		-0.001	0.991	
Employment statues	-0.145	**0.030**		-0.171	**0.009**	
Work Experience	-0.073	0.241		-0.033	0.589	
Workplace	0.080	0.262		0.104	0.136	
Educational levels	0.048	0.434		0.054	0.365	
Number of Missions in 24 h	-0.086	0.246		-0.039	0.589	
Type of shift	-0.154	**0.028**		-0.086	0.203	
Overtime hours per month	0.130	**0.036**		0.104	0.084	

The results showed that variables of physical fatigue (*p* = 0.022), the partner trust and shared mental models (PTSMM) (*p* = 0.001), age (*p* = 0.024), and employment status (*p* = 0.030), type of shift (*p* = 0.028), and overtime hours per month (*p* = 0.036) were significant predictors of general unsafe behavior, the partner trust and shared mental models (PTSMM) (*p* = 0.019), the mild task conflict (MTC) (*p* = 0.001), and employment status (*p* = 0.009) were significant predictors of unsafe behavior under incentives.

## Discussion

Unsafe behaviors among EMS staff are considered significant and critical social issues. They can adversely affect patients and staff, potentially impeding healthcare service delivery. This study aimed to assess the status of unsafe behaviors and their relationship with work-related factors among EMS staff.

The study results showed that the average score of unsafe behaviors of EMS staff is 15.80 (± 4.77). Unsafe behaviors of the participants were mainly related to general unsafe behaviors (ignoring safety regulations, breaking work procedures, taking chances, bending the rules, ignoring some rules, stopping working to the rules due to workplace conditions, and carrying out forbidden activities). It was also found that the participants were less inclined to engage in unsafe behaviors under incentives (breaking the rules due to incentives, breaking the rules due to management pressure, breaking the rules due to coworker provocation). In a study conducted by Sedlar with Slovakian emergency medical personnel, it was found that their average score of unsafe behaviors was higher compared to the results of our study [11]. This suggests that there are various factors that may influence the score of unsafe behaviors, such as the cultural differences between countries and the individual differences of emergency medical workers that can affect their decision-making and behavior [11, 43]. Other similar studies [5, 26, 35] showed that whatever employees report more unsafe behaviors, safety incidents will be more frequent in that profession. This indicates that compliance with the rules, work procedures, and safety regulations is appropriate to prevent the risk of harming the health of oneself, coworkers, or patients. Some EMS conditions, such as saving patients’ lives, unfortunately, lead to haste and unsafe behaviors of the staff. Therefore, adopting a more rational and cautious approach to follow the rules is recommended.

This study also identified partner trust and shared mental models (PTSMM) as predictors of general unsafe behavior and unsafe behavior under incentives among EMS staff. It was found that an increase in PTSMM is associated with a decrease in unsafe behaviors, indicating its significance in promoting a safer work environment among EMS staff. This result is consistent with previous studies [35, 44], which have shown that teamwork is a practical approach to improve the treatment process and reduce errors. EMS managers can prioritize initiatives to strengthen partner trust and shared mental models within the EMS setting.

Mild task conflict was one of the predictors of unsafe behavior under incentives. The findings of the Glawing et al. study showed that promoting cooperation is essential to ensuring high-quality and safe care [18]. Khoshab et al. study also showed that teamwork is related to the team’s work factors, and the personality characteristics of the members will have the most significant impact on the team’s behavioral achievements [28]. According to similar studies, the presence of competent colleagues, fostering cooperation in the team, can significantly improve the care provided and increase safety. It is also significantly associated with lower occurrences of adverse events [30, 32]. EMS managers can improve staff EMS’ teamwork competency by considering multiple educational strategies, including clarifying roles and responsibilities, managing conflict, and structured teamwork training.

Based on the results of this study, physical fatigue, the type of shift, and overtime hours were predictors of general unsafe behaviors. Participants who reported 24-hour shifts had more general unsafe behaviors. This result was consistent with Donnelly et al. study, which showed that paramedics with higher fatigue levels had more safety-compromising behaviors [17]. Previous research is also consistent with the present study and shows that fatigue caused by shift work and long hours is a risk to safety in health care [23, 26, 45]. The results of Weaver et al. showed that extended weekly work hours were unrelated to EMS worker fatigue [46]. According to these results, with low fatigue levels, EMS staff may be more focused on their tasks and less engaged in unsafe behaviors. Managers and health organizations should pay attention to their personnel’s physical and mental health and implement measures to reduce fatigue and increase safety in the workplace.

Another predictor of general unsafe behaviors was the age of the participants; younger staff showed less desire to perform safe behaviors than older staff. In a similar study, younger paramedics reported more safety-compromising behaviors than older paramedics [17]. In another study, changes in safety culture scores were not affected by age, which could be related to the culture, gender, or work experience of personnel [45]. This result may be explained by the older staff’s higher experience, job security, and work stability, which reduces their risk-taking tendency and increases their obedience to safety rules and procedures.

Employment status was also significantly related to general unsafe behavior and unsafe behavior under incentives. The staff with a Commitment status reported higher rates of unsafe behaviors, consistent with the results of Kosydar et al. study [45]. It seems that staff with an employed status can control themselves in different situations and have better performance and less unsafe behaviors. It is recommended to use the experience and professional levels of EMS staff to reduce unsafe behaviors.

## Limitations

This study can be the basis for further research regarding unsafe behaviors in Iran’s healthcare setting. The study involved EMS staff from all urban and roadside bases, which makes the results helpful in policy-making in Ardabil province. However, the present study had some limitations. First, the findings are from a selected geographical location in Ardabil, Iran. Therefore, our results should be generalized with caution. Second, the present study measured variables using only self-reported questionnaires, which, in turn, can expose the results to bias. Third, the study was conducted using cross-sectional method. Longitudinal studies can provide more information in the future.

## Conclusion

The present study showed that “partner trust and shared mental models (PTSMM),” “mild task conflict (MTC),” “physical fatigue,” “type of shift,” “overtime hours per month,” “age,” and “employment status,” were predictors of unsafe behaviors. Extended programs should be considered due to the negative consequences of unsafe behaviors. Developing specific guidelines for addressing unsafe behaviors and reducing these behaviors among EMS staff can be beneficial. EMS managers should pay attention to overtime hours in the workplace and implement measures to reduce staff fatigue. They can improve teamwork by considering multiple educational strategies, including clarifying roles and responsibilities. Establishing a system where novice staff work with experienced staff during their first year can enhance safety, reduce unsafe behaviors, and foster trust and shared mental models among team members.

## Data Availability

The datasets used and analyzed during the current study are available from the corresponding author upon reasonable request.
